# UV-Enhanced Humidity Sensing of Chitosan–SnO_2_ Hybrid Nanowires

**DOI:** 10.3390/nano10020329

**Published:** 2020-02-14

**Authors:** Orhan Sisman, Navpreet Kaur, Giorgio Sberveglieri, Estefania Núñez-Carmona, Veronica Sberveglieri, Elisabetta Comini

**Affiliations:** 1 Sensor Lab, Department of Information Engineering, University of Brescia, 25123 Brescia, Italy; o.sisman@unibs.it (O.S.); n.kaur001@unibs.it (N.K.); giorgio.sberveglieri@unibs.it (G.S.); 2 Nano Sensor Systems S. r. l., 25123 Brescia, Italy; 3 CNR-IBBR Institute of Biosciences and Bioresources, 50019 Florence, Italy; estefania.nunezcarmona@ibbr.cnr.it (E.N.-C.); veronica.sberveglieri@unibs.it (V.S.)

**Keywords:** chitosan, SnO_2_, hybrid, nanostructures, room temperature, humidity sensor

## Abstract

The surface of SnO_2_ nanowires was functionalized by chitosan for the development of room-temperature conductometric humidity sensors. SnO_2_ nanowires were synthesized by the seed-mediated physical-vapor-deposition (PVD) method. Chitosan layers were deposited on top of the SnO_2_ nanowires by spin coating. Surface morphology, crystal structure, and optical properties of the synthesized hybrid nanostructure were investigated by scanning electron microscope, grazing incidence X-ray diffraction, and UV–Vis absorption measurements. During electrical conductivity measurements, the hybrid nanostructure showed unusual behavior towards various relative humidity (RH) concentrations (25%, 50%, 75%), under UV-light irradiation, and in dark conditions. The highest sensor responses were recorded towards an RH level of 75%, resulting in 1.1 in the dark and 2.5 in a UV-irradiated chamber. A novel conduction mechanism of hybrid nanowires is discussed in detail by comparing the sensing performances of chitosan film, SnO_2_ nanowires, and chitosan@SnO_2_ hybrid nanostructures.

## 1. Introduction

Organic conductive polymers are used in many varied applications [1,2,3]. Due to their extended π-conjugation of the polymer chain by alternating double bond–single bond structure, partial oxidation causes variations in electrical conduction with the formation of charges on their chains [4]. In this way, some, including polyaniline (PAni), polypyrrole (PPy), and polythiophene (Pth), form chemoresistive surfaces with their special molecular structures [5,6,7]. They alter their electrical conductance when they are exposed to some gases. They have many advantages: ease of construction, optimum performance at room temperature, low power consumption, rapid and reversible adsorption/desorption kinetics, low toxicity, and high dependency on analyte concentration compared to inorganic materials [8].

Chitosan is a well-known polymer formed by repeating units of 1,4-linked 2-deoxy-2-amine glucose. It has noteworthy properties such as film-forming ability, biocompatibility, biodegradability, hydrophilicity, and, importantly, non-toxicity. The main feature that makes it favorable is the use of chitin, one of the most abundant polysaccharides in nature, as source material [9]. Many studies are currently working on chitosan-derivative products because of their sustainable, eco-friendly, and economic benefits. 

It is an insulating polymer, but protonated amine groups in its chains make it conductive and sensitive to polar gas vapors. Therefore, metals, carbon nanotubes, metal oxides, and conductive polymers mixed with chitosan layers have been tested as gas-sensitive materials. For instance, Mironenko et al. studied the changes in optical waveguides made of Ag nanoparticles mixed chitosan and Au nanoparticles mixed chitosan composites in the presence of H_2_S gas [10]. Abu-Hani et al. fabricated flexible H_2_S sensing films by adding glycerol to chitosan polymers [11]. Kumar et al. discovered the chemo-electrical behavior of carbon nanotube (CNT) blended chitosan layers towards water, methanol, and toluene vapors [12]. Li et al. synthesized PAni–chitosan nanocomposites for hydrogen sensors [13]. Ayad et al. revealed dimethylamine sensing properties of chitosan blended polyaniline nanofibers [14]. Similarly, Wang et al. decorated chitosan nanofibers with polyethyleneimine to detect formaldehyde [15]. In most of these studies, chitosan has been used as a film-forming material, not as an active sensing material. In addition to these, chitosan was used in more complex composites for gas sensing [16,17]. Molla-Abbasi and Ghaffarian suggested the use of chitosan-decorated carbon nanotube surfaces for polar chemical vapors [18]. They discovered improved humidity sensing abilities of chitosan@CNT heterostructures related with the chitosan surfaces. Dai et al. also verified these observations by presenting the humidity sensing properties of chitosan-decorated ZnO–SWCNT (single-walled carbon nanotube) structures [19]. Xu et al. increased the humidity sensing abilities of bare ZnO nanorods by four times by using a chitosan layer on top of ZnO nanorods [20].

To date, the research has focused on the usage of chitosan to get an advantage from its hydrophilic character. A hybrid structure of chitosan and SnO_2_ nanowires (NWs) was proposed for a conductometric humidity sensor using a similar approach, as tin oxide (SnO_2_) has been the most attractive sensing material with its tailor-made semiconducting properties [21]. Its charge carriers have a high concentration and high mobility at elevated temperatures [21,22]. Considering the instability of chitosan at high temperature, UV light irradiation was used to benefit from the semiconducting properties of SnO_2_ NWs.

## 2. Materials and Methods

### 2.1. Synthesis of SnO_2_ Nanowires

A seed-layer-assisted vapor–liquid–solid (VLS) technique was used for the synthesis of SnO_2_ NWs. Pre-cleaned alumina substrates (3 × 3 mm^2^) were coated with an Au seed layer by sputtering at a 6.6 × 10^−3^ mbar working pressure and 50 W RF magnetron power (Ar plasma) for 5 s. The VLS growth was carried out in a physical-vapor-deposition (PVD) furnace at 100 mbar (Ar flow of 100 sccm) for 2 min. The SnO_2_ powder was placed in the middle of the furnace and heated up to 1370 °C, while the substrates were placed at a small distance from SnO_2_ dust at a temperature of 860 °C.

### 2.2. Synthesis of Chitosan@SnO_2_ Hybrid Nanowires

A chitosan solution was prepared by dissolving 0.3 g low-molecular-weight chitosan powder (Sigma-Aldrich, Darmstadt, Germany, CAS no: 9012-76-4) in acetic acid aqueous solution (1.5%). Then, the chitosan solution was deposited on top of SnO_2_ nanowires by spin coating. A 50 µL aliquot of the prepared solution was dripped onto rotating samples at 6000 rpm. The spinning time lasted 45 s after two drops. Then, samples were taken into a clean place to allow for drying of the layer for 48 h.

### 2.3. Sample Characterization

The formation of SnO_2_ nanowires and the deposition of chitosan layers were monitored by a field-emission scanning electron microscope (FE-SEM, Leo 1525 Gemini model; Carl Zeiss AG, Oberkochen, Germany). Surface images of chitosan@SnO_2_ nanowires were taken under small potential ranges (1–5 kV) and in tilted positions (45° and 75°) to avoid damaging the chitosan layers. The crystalline properties of SnO_2_ were demonstrated by X-ray diffractometry (Empyrean; PANalytical, Almelo, Netherlands) measurements in glancing angle mode (with an incident angle of 1.5°). A Cu-LFF (λ = 1.5406 Å tube was used for X-ray emissions and operated at 40 kV/40 mA. The spectra were recorded by using a Xe detector in the range of 20–60°. Optical properties of SnO_2_ and chitosan@SnO_2_ nanowires were determined by a UV-vis spectrometer (UV-2600, Shimadzu, Kyoto, Japan).

### 2.4. Conductometric Humidity Measurements 

**Figure 1:**
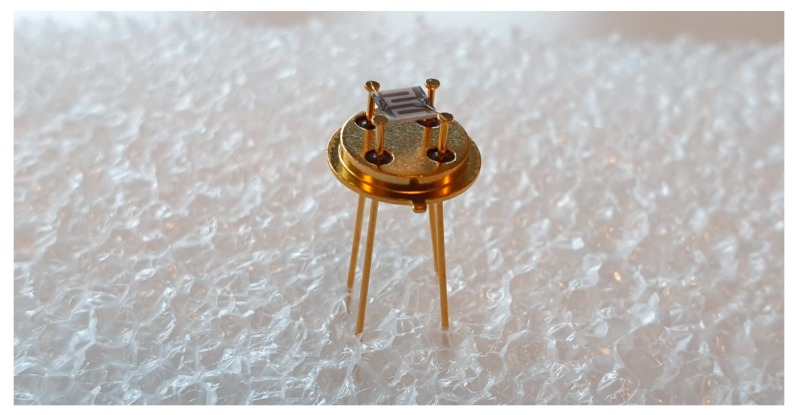
An image of a chitosan@SnO_2_ hybrid nanowire (NW) sensor.

Transducers with SnO_2_ and chitosan@SnO_2_ nanowires were equipped with contacts by magnetron sputtering to carry out electrical measurements. The TiW and Pt contact pads were deposited at 75 W DC power (Ar plasma) and 6.6 × 10^−3^ mbar working pressure for 3 min respectively in order to get adhesion. Then, a Pt interdigitated transducer (IDT) was deposited under the same pressure and DC power conditions for 20 min (≈1 μm thickness). The sensors, integrated into TO-8 cases, were placed in a climatic test chamber for humidity measurements. An integrated hybrid sensor is shown below in Figure 1.

The conductometric changes were monitored by picoammeter during humid air injections and dry air flows. The tested relative humidity (RH) concentrations were 25%, 50%, 75%, 50%, and 25% respectively. The measurement was repeated in the same conditions under UV light irradiation. The UV light source had a ~365 nm wavelength (λ_max_); however, a 350 nm Dichroic filter (T = 30%) was used during the measurement to control the irradiation power of 0.5 µW/mm^2^. The response values were calculated according to conduction type (n- or p-) of chitosan and hybrid nanowires using the following formulae:

(1) $$Response\;\left(R\right)=\frac{G_{air}-G_{gas}}{G_{gas}}\;\left(n-type\right)$$

(2) $$Response\;\left(R\right)=\frac{G_{gas}-G_{air}}{G_{air}}\;\left(p-type\right)$$

## 3. Results and Discussion

### 3.1. Material Characterizations

**Figure 2:**
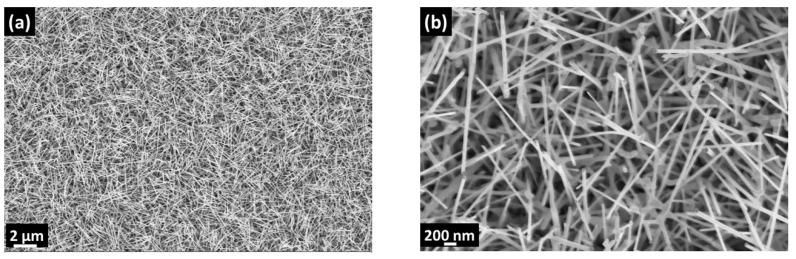
(**a**) 10k and (**b**) 50k × Magnified SEM images of SnO_2_ nanowires.

The formation of SnO_2_ nanowires followed the VLS mechanism. The Au nano-seed layer became liquid at 860 °C @100 mbar. The SnO_2_ powder evaporated and the Ar flow acted as a gas carrier to transport SnO_2_ vapors from the source to the substrates. The formation of SnO_2_ nanowires was triggered by the condensation of the SnO_2_ vapors on top of the Au seed layers and by the ensuing segregation at the bottom of the droplet. The fabricated SnO_2_ nanowires were homogenous through the substrate surface (Figure 2). They were 2–4 µm long and 50–80 nm thick.

**Figure 3:**
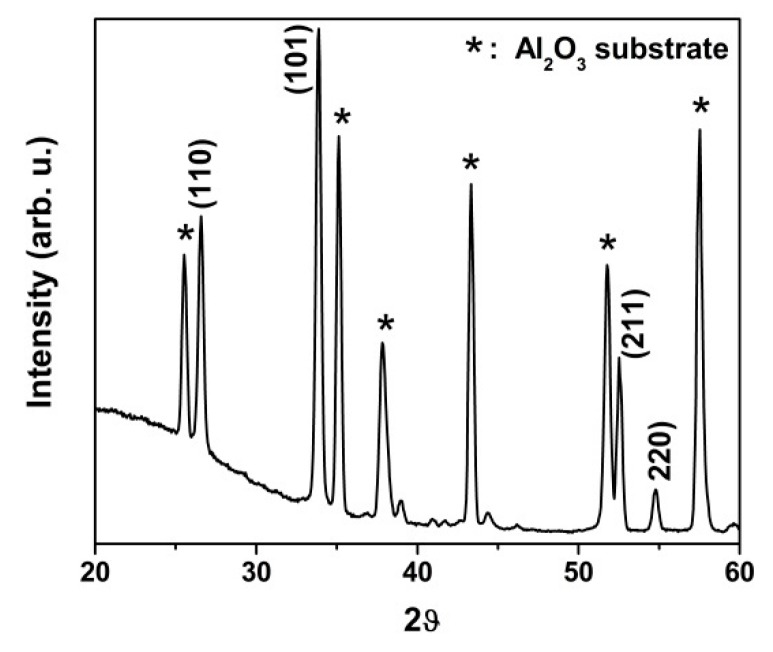
GI-XRD profile of SnO_2_ nanowires.

The GI-XRD profile of SnO_2_ nanowires is shown in Figure 3. The characteristic peaks of SnO_2_ nanowires belonged to (110), (101), (211), and (220) crystalline planes located at 26.8°, 33.3°, 52.2°, and 54.8°, respectively. The rutile crystalline phase of SnO_2_ nanowires was consistent with the standard JCPDS 41-1445 pattern. The polycrystalline alumina substrate peaks were marked with “*” in the GI-XRD spectrum. The small quantity of Au peaks, around 38° and 44°, displayed the low presence of Au traces. It is a common mechanism of VLS that the catalyst (Au) comes up on the top of nanowires (also visible in Figure 2b).

**Figure 4:**
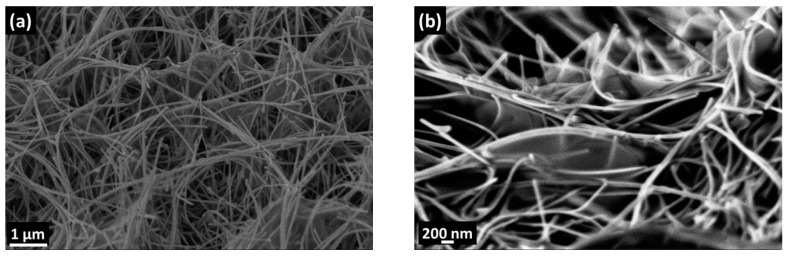
SEM images of chitosan-deposited SnO_2_ nanowires (**a**) 45° tilted and (**b**) 75° tilted surfaces.

Despite the low viscosity of the chitosan solution, the chitosan layers were successfully deposited by spin coating. At 6000 rpm rotation rates, the drop of chitosan spread through the surface of SnO_2_ NWs. The surface morphologies of the chitosan@SnO_2_ NWs hybrid (Figure 4) were investigated by SEM at 45° (a) and 75° (b) tilted positions (Figure 4). Chitosan layers could be seen as blurred sheets connecting the nanowires. They did not cover the surface of nanowires completely but filled the empty spaces between nanowires.

**Figure 5:**
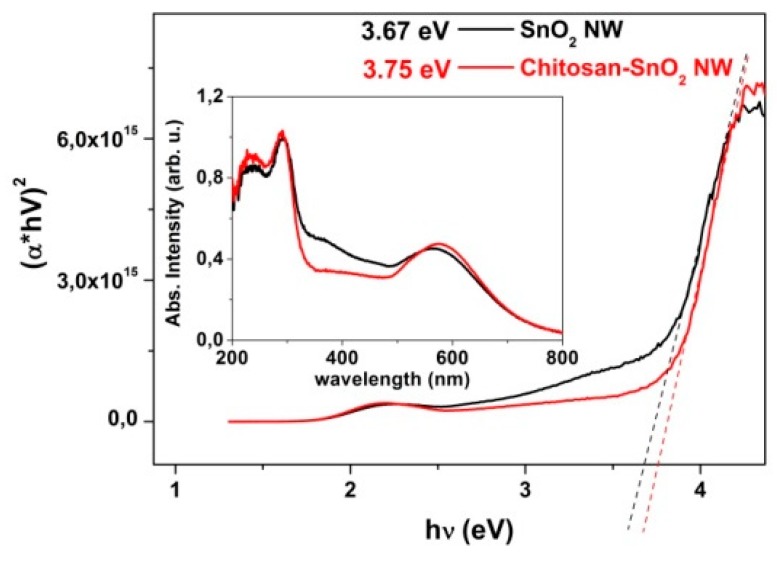
Tauc’s plot and UV–Vis absorbance spectra.

The UV–Vis absorption profile (inset in Figure 5) was used to determine the optical band gap and the type of band-to-band transition of the SnO_2_ NWs and chitosan@SnO_2_ NWs hybrid. We plotted the (αhν)^2^ versus hν in Figure 5 using Tauc’s method. The optical band gap energy values of SnO_2_ and chitosan@SnO_2_ NWs were estimated as 3.67 and 3.75 eV, respectively. The optical band gap energy value of SnO_2_ NWs was consistent with the literature value (3.6 eV). Both plots demonstrate that there was a limited effect of the chitosan layer on the absorption of UV light. Even so, chitosan has been used in many optical sensor systems based on transparency change by blending functional materials [23,24,25,26].

### 3.2. Humidity Sensing

**Figure 6:**
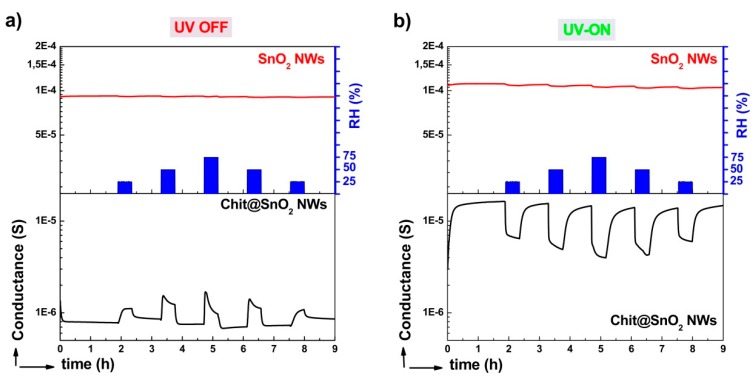
Dynamic sensor responses of bare SnO_2_ and chitosan@SnO_2_ hybrid NWs in the dark (**a**) and under UV light irradiation (**b**).

**Figure 7:**
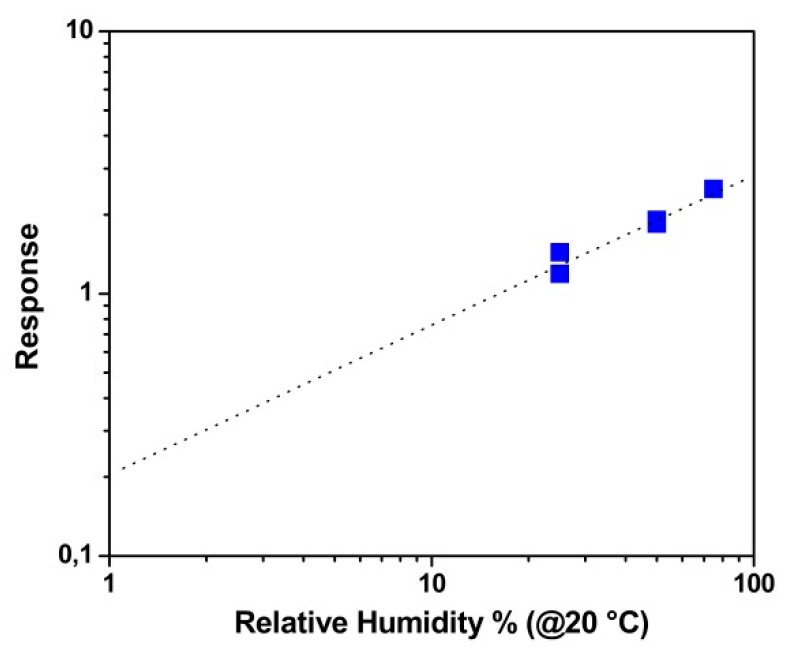
Response values of hybrid nanowires under UV light vs. measured relative humidity (RH) levels in logarithmic scales.

Figure 6 reports the dynamic sensor responses of both the SnO_2_ and chitosan@SnO_2_ hybrid NWs towards humidity changes in the dark (a) and UV-light-irradiated (b) chambers. The baseline value of bare SnO_2_ NWs was measured at around 1 × 10^−4^ S, drawn in red. SnO_2_ NWs did not exhibit any significant changes towards water vapor injections in either measurement. However, the baseline conductance value of chitosan@SnO_2_ hybrid NWs increased approximately by one order of magnitude, from 8 × 10^−7^ S to 1.5 × 10^−5^ S, under UV light. A similar change was observed with the conductance measurement of ZnO thin film coated bacterial cellulose membranes in previous work [27]. In the dark chamber, humidity changes led to variations in the response profiles of chitosan@SnO_2_. The conductance value increased exponentially at the level of 25% RH. Additionally, it decreased exponentially after a rapid increase at 50% and 75% RH levels. The maximum response values were recorded as 1.1 in the dark, and 2.5 under UV-light-irradiated measurements both towards 75% RH (at 20 °C). The detection limit of the hybrid sensor was determined as 5% RH for the UV-irradiated measurement by using the calibration curve (dotted line) as seen in Figure 7 and by considering 0.5 as the minimum significant response value.

**Figure 8:**
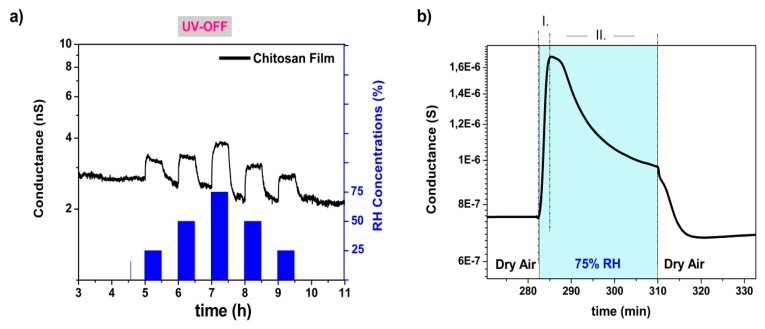
(**a**) Humidity measurement profile of the chitosan film. (**b**) Zoomed conductance profile of chitosan@SnO_2_ nanowires hybrid in dark condition towards 75% RH (at 20 °C) injection.

Figure 8a reports the humidity-sensing capabilities of the chitosan film on an alumina substrate. Despite its lower surface area and its lower conductivity, the chitosan film showed sensor signals proportional to humid air concentrations. The conductance value increased by the injections of moist air, similar to the dark measurement profile of chitosan@SnO_2_ hybrid nanowires. As can be easily deduced by comparing the humidity-sensing performances of bare SnO_2_ and bare chitosan film, chitosan was the main actor in the humidity sensing of hybrid NWs. In previous studies, the sensing mechanism of chitosan was explained by the proton-conduction mechanism [28,29]. The adsorbed water molecules on the surface cause neutralization of protonated amine groups in chitosan chains. The primary electrochemical process on the chitosan surface has been described by the following equation [29]:

(3) $$R\ldots {\mathrm{NH}}_{3}^{+}\overset{H_{2}O}{\to }R\ldots {\mathrm{NH}}_{2}+H^{+}$$

**Figure 9:**
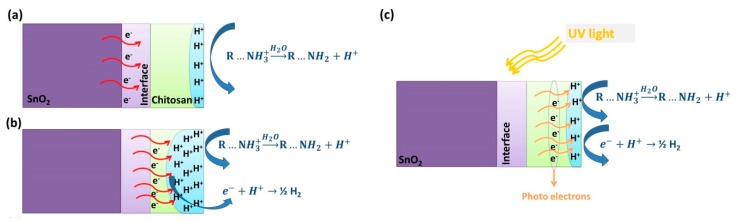
The humidity-sensing mechanism of hybrid chitosan@SnO_2_ nanowires (**a**) in a low level of humidity, (**b**) in a high level of humidity, and (**c**) under UV irradiation.

The dynamic measurement data of the hybrid sensor were plotted on a small scale in Figure 8b. In I and II regions, indicated by dashed lines in Figure 8b, the hybrid sensor had dual conduction behavior under a constant humid air concentration (75% RH). In region I, the conductance value increased suddenly after water vapor injection. The electrical conduction of the hybrid nanostructures was dominated by protonic conduction, as given before in Equation (3). The protonic conduction reduced the resistance of the chitosan layer rapidly and caused a decrease in the equivalent resistance of the hybrid system. In region II, the conductance started to decrease after reaching a peak point during the constant flow of water vapor. It was an interesting observation since the bare SnO_2_ sample did not show any significant response to water vapor injections. The only possible explanation could be that the valance electrons of SnO_2_ participated in the surface reactions and sensing mechanism. Rana et al. highlighted a change in the dielectric environment after the absorption of vapor molecules on a CNT–chitosan surface [30]. Similarly, the protonic conduction of chitosan might create an electrical potential at the chitosan–SnO_2_ interface. The $H^{+}$ agglomeration on the surface attracted the valance electrons of SnO_2_ and caused an increase in the equivalent resistance of the hybrid system, as shown in Figure 9b. The attracted valance electrons neutralized the agglomerated $H^{+}$ ions and reduced the protonic conduction, as given below in Equation (4) [31]:

(4) $$e^{-}+H^{+}\to \frac{1}{2}H_{2}$$

The dual conduction was not observed in 25% RH. This observation indicates that there is a threshold RH value between 25–50% RH to activate valance electrons of SnO_2_.

### 3.3. UV Effect on Sensing

There was no significant effect of UV light on the dynamic measurement profile of bare SnO_2_ NWs, as seen in Figure 6b. Besides, the measurement profile of the chitosan@SnO_2_ hybrid NWs was reversed from p-type to n-type under UV illumination. Additionally, the response values of the hybrid sample were increased compared to dark measurements. Even though SnO_2_ was the active material for UV absorption, proven by the optical analysis above, hybrid nanocomposite sensors showed better performance compared to bare SnO_2_. The UV light irradiation generated electron–hole pairs in the SnO_2_ surface. In the literature, some studies proposed that SnO_2_ and its heterostructure showed better NO_2_ detection under UV light because generated photoelectrons captured more O_2_ molecules on the surface, resulting in band bending [32,33,34]. Similarly, in the present case, the UV light may have bent the potential energy barrier between chitosan and SnO_2_ NWs. Thus, valance electrons could take place in the surface reactions. This proposed mechanism explains why hybrid sensors reversed the conduction type under UV irradiation and why there were no dual conduction behaviors at levels of 50% and 75% RH as observed in the dark measurement profiles.

Table 1 summarizes the response values of the chitosan@SnO_2_ hybrid NWs and some other chemoresistor-type chitosan-based humidity detectors. A direct comparison of response values cannot be fair for the performance evaluation since the reported studies applied different test procedures and defined response parameters with different methodologies. For example, in two of the studies, response values were fitted by using the Langmuir–Henry–Clustering (LHC) model to correlate gas sensing with properties of CNT–chitosan and polyvinly alcohol (PVA)–chitosan–CNT blended films [12,18]. Thus, they used nitrogen as a carrier gas during their measurements. One study has reported the best response value with chitosan films deposited by spin coating on a quartz substrate [29]. However, the baseline conductance value of chitosan film was around 0.1 nS, which is challenging for real applications. After considering differences between previous studies, our hybrid sensors exhibited satisfactory and more practical performance.

## 4. Conclusions

SnO_2_ NWs were combined with chitosan layers to take advantage of the hydrophilic property of chitosan for a room-temperature humidity detector. The study aimed to increase the surface area of the chitosan layer and to get biocompatible sensing layers and transducer material. Chitosan polymer was deposited by a spin-coating method on top of SnO_2_ NWs. The humidity sensing of the chitosan@SnO_2_ NWs hybrid showed better sensing properties than both chitosan thin films and bare SnO_2_ NWs. The dual conduction behavior of hybrid NWs was explained with a new mechanism combining the protonic conduction and the effect of the chitosan@SnO_2_ NWs hybrid. The surface reactions, possible chemical interactions, and effects of UV irradiation were discussed, and a sensing mechanism was proposed. Chitosan@SnO_2_ hybrid NWs exhibited better sensing performances under UV light irradiation.

## Figures and Tables

**Table 1:** Comparison table of chitosan-based conductometric humidity sensors.

Material	Transducer	RH Conc.	Response	Reference
2% CNT-Chitosan	Chemoresistor	1% Vapor Conc.	1	[12]
PVA/Chitosan/CNT	Chemoresistor	10 ‰Vapor	0.05	[18]
Chitosan/ZnO/SWCNT	Chemoresistor	75%	0.75	[19]
Chitosan	Chemoresistor	α% RH	4.2 × α	[29]
Chitosan@SnO_2_ Hybrid Nanowires	Chemoresistor	75% RH (dark)	1.1	This work
		75% RH (UV irradiation)	2.5	This work

## References

[1] Li D., Lai W.Y., Zhang Y.Z., Huang W. (2018). Printable Transparent Conductive Films for Flexible Electronics. Adv. Mater..

[2] Bharti M., Singh A., Samanta S., Aswal D.K. (2018). Conductive polymers for thermoelectric power generation. Prog. Mater. Sci..

[3] Kim F.S., Ren G., Jenekhe S.A. (2011). One-dimensional nanostructures of π-conjugated molecular systems: Assembly, properties, and applications from photovoltaics, sensors, and nanophotonics to nanoelectronics. Chem. Mater..

[4] Bailey R.A., Persaud K.C., Osada Y., De Rossi D.E. (2000). Sensing Volatile Chemicals Using Conducting Polymer Arrays. Polymer Sensors and Actuators.

[5] Huang J., Virji S., Weiller B.H., Kaner R.B. (2004). Nanostructured Polyaniline Sensors. Chem. A Eur. J..

[6] Li B., Santhanam S., Schultz L., Jeffries-EL M., Iovu M.C., Sauvé G., Cooper J., Zhang R., Revelli J.C., Kusne A.G. (2007). Inkjet printed chemical sensor array based on polythiophene conductive polymers. Sens. Actuators B Chem..

[7] Ameer Q., Adeloju S.B. (2005). Polypyrrole-based electronic noses for environmental and industrial analysis. Sens. Actuators B Chem..

[8] Bai H., Shi G. (2007). Gas Sensors Based on Conducting Polymers. Sensors.

[9] Nunes C., Coimbra M.A., Ferreira P. (2018). Tailoring Functional Chitosan-Based Composites for Food Applications. Chem. Rec..

[10] Mironenko A.Y., Sergeev A.A., Nazirov A.E., Modin E.B., Voznesenskiy S.S., Bratskaya S.Y. (2016). H2S optical waveguide gas sensors based on chitosan/Au and chitosan/Ag nanocomposites. Sens. Actuators B Chem..

[11] Abu-Hani A.F.S., Greish Y.E., Mahmoud S.T., Awwad F., Ayesh A.I. (2017). Low-temperature and fast response H2S gas sensor using semiconducting chitosan film. Sens. Actuators B Chem..

[12] Kumar B., Feller J.F., Castro M., Lu J. (2010). Conductive bio-Polymer nano-Composites (CPC): Chitosan-carbon nanotube transducers assembled via spray layer-by-layer for volatile organic compound sensing. Talanta.

[13] Li W., Jang D.M., An S.Y., Kim D., Hong S.K., Kim H. (2011). Polyaniline-chitosan nanocomposite: High performance hydrogen sensor from new principle. Sens. Actuators B Chem..

[14] Ayad M.M., Salahuddin N.A., Minisy I.M., Amer W.A. (2014). Chitosan/polyaniline nanofibers coating on the quartz crystal microbalance electrode for gas sensing. Sens. Actuators B Chem..

[15] Wang N., Wang X., Jia Y., Li X., Yu J., Ding B. (2014). Electrospun nanofibrous chitosan membranes modified with polyethyleneimine for formaldehyde detection. Carbohydr. Polym..

[16] Tang Q., Shi X., Hou I., Zhou J., Xu Z. (2014). Development of molecularly imprinted electrochemical sensors based on Fe3O4@MWNT-COOH/CS nanocomposite layers for detecting traces of acephate and trichlorfon. Analyst.

[17] Hua E., Wang L., Jing X., Chen C., Xie G. (2013). One-step fabrication of integrated disposable biosensor based on ADH/NAD+/meldola’s blue/graphitized mesoporous carbons/chitosan nanobiocomposite for ethanol detection. Talanta.

[18] Molla-Abbasi P., Ghaffarian S.R. (2014). Decoration of carbon nanotubes by chitosan in a nanohybrid conductive polymer composite for detection of polar vapours. RSC Adv..

[19] Dai H., Feng N., Li J., Zhang J., Li W. (2019). Chemiresistive humidity sensor based on chitosan/zinc oxide/single-walled carbon nanotube composite film. Sens. Actuators B Chem..

[20] Xu J., Bertke M., Li X., Mu H., Zhou H., Yu F., Hamdana G., Schmidt A., Bremers H., Peiner E. (2018). Fabrication of ZnO nanorods and Chitosan@ZnO nanorods on MEMS piezoresistive self-actuating silicon microcantilever for humidity sensing. Sens. Actuators B Chem..

[21] Kim H.J., Lee J.H. (2014). Highly sensitive and selective gas sensors using p-type oxide semiconductors: Overview. Sens. Actuators B Chem..

[22] Li R., Zhou Y., Sun M., Gong Z., Guo Y., Wu F., Li W., Ding W. (2019). Influence of Charge Carriers Concentration and Mobility on the Gas Sensing Behavior of Tin Dioxide Thin Films. Coatings.

[23] Dykstra P., Hao J., Koev S.T., Payne G.F., Yu L., Ghodssi R. (2009). An optical MEMS sensor utilizing a chitosan film for catechol detection. Sens. Actuators B Chem..

[24] Abdullah J., Ahmad M., Lee Y.H., Karuppiah N., Sidek H. (2007). An optical biosensor based on immobilization of lacease and MBTH in stacked films for the detection of catechol. Sensors.

[25] Powers M.A., Koev S.T., Schleunitz A., Yi H., Hodzic V., Bentley W.E., Payne G.F., Rubloff G.W., Ghodssi R. (2005). A fabrication platform for electrically mediated optically active biofunctionalized sites in BioMEMS. Lab Chip.

[26] Koev S.T., Dykstra P.H., Luo X., Rubloff G.W., Bentley W.E., Payne G.F., Ghodssi R. (2010). Chitosan: An integrative biomaterial for lab-on-a-chip devices. Lab Chip.

[27] Núñez-Carmona E., Bertuna A., Abbatangelo M., Sberveglieri V., Comini E., Sberveglieri G. (2019). BC-MOS: The novel bacterial cellulose based MOS gas sensors. Mater. Lett..

[28] Bouvree A., Feller J.F., Castro M., Grohens Y., Rinaudo M. (2009). Conductive Polymer nano-bioComposites (CPC): Chitosan-carbon nanoparticle a good candidate to design polar vapour sensors. Sens. Actuators B Chem..

[29] Zou J., Zhang K., Zhang Q. (2016). Giant Humidity Response Using a Chitosan-Based Protonic Conductive Sensor. IEEE Sensors J..

[30] Rana V.K., Akhtar S., Chatterjee S., Mishra S., Singh R.P., Ha C.S. (2014). Chitosan and Chitosan-co-Poly(-caprolactone) Grafted Multiwalled Carbon Nanotube Transducers for Vapor Sensing. J. Nanosci. Nanotechnol..

[31] Yi H., Wu L.Q., Bentley W.E., Ghodssi R., Rubloff G.W., Culver J.N., Payne G.F. (2005). Biofabrication with chitosan. Biomacromolecules.

[32] Park S., An S., Mun Y., Lee C. (2013). UV-enhanced NO_2_ gas sensing properties of SnO_2_-Core/ZnO-shell nanowires at room temperature. ACS Appl. Mater. Interfaces.

[33] Li J., Gu D., Yang Y., Du H., Li X. (2019). UV Light Activated SnO2/ZnO Nanofibers for Gas Sensing at Room Temperature. Front. Mater..

[34] Prades J.D., Jimenez-Diaz R., Manzanares M., Hernandez-Ramirez F., Cirera A., Romano-Rodriguez A., Mathur S., Morante J.R. (2009). A model for the response towards oxidizing gases of photoactivated sensors based on individual SnO_2_ nanowires. Phys. Chem. Chem. Phys..

